# Automated synthesis of [^11^C]L-glutamine on Synthra HCN plus synthesis module

**DOI:** 10.1186/s41181-019-0057-4

**Published:** 2019-03-20

**Authors:** Prashanth K. Padakanti, Shihong Li, Alexander Schmitz, David Mankoff, Robert H. Mach, Hsiaoju S. Lee

**Affiliations:** Department of Radiology, University of Pennsylvania, Philadelphia, PA 19104 USA

**Keywords:** [^11^C]L-glutamine, Radiochemical synthesis, Synthra HCN plus module and HPLC

## Abstract

**Background:**

L-Glutamine (L-Gln) is the most abundant amino acid present in the human body and is involved in numerous metabolic pathways. Glutaminolysis is the metabolic process deployed by many aggressive cancers such as triple negative breast cancer (TNBC). Imaging the metabolic pathways of L-glutamine could provide more insights into tumor biology. Reliable and reproducible automated synthesis of [^11^C]L-glutamine PET (Positron Emission Tomography) radiotracer is critical for these studies.

**Results:**

[^11^C]L-Glutamine ([^11^C]L-Gln) was reliably and reproducibly synthesized. The automated process involves cleaning and drying of the synthesis module, azeotropic drying of crown ether and cesium bicarbonate, conversion of [^11^C]CO_2_ to [^11^C] CsCN, incorporation of [^11^C] CN into the starting material, and hydrolysis and deprotection of the corresponding [^11^C] nitrile to yield [^11^C]L-glutamine. Starting with approximately 1 Ci of [^11^C] cesium cyanide ([^11^C]CsCN), 47–77 mCi (*n* = 4) of the final product, [^11^C]L-Gln, was obtained after sterile filtration. The radiochemical purity of the final product was > 90% with almost exclusively L-glutamine isomer. The yield of [^11^C]L-Gln was 43–52% (n = 4), decay corrected to end of [^11^C] CsCN trapping in the reaction vessel.

**Conclusions:**

All the steps including drying of the mixture of base and crown ether, preparation of [^11^C] cyanide, radiochemical synthesis and formulation were accomplished on a single synthesis unit. [^11^C]L-Gln has been successfully adapted and optimized on an automated synthesis module, Synthra HCN Plus. This process can be readily adapted for clinical research use.

## Background

L-Glutamine (L-Gln), the most abundant amino acid present in the human body is involved in numerous metabolic processes. (Bergstrom et al., 1974; Sai et al., 2017) In some cancer cells, glutamine metabolism can be altered; the oncogene, Myc, upregulates mitochondrial glutaminase, which activates glutaminolysis. (Wise et al., 2008) Glutaminolysis is a metabolic pathway exploited by many aggressive cancers, including triple negative breast cancers (TNBC), as a means to utilize glutamine for both energy metabolism and biosynthesis, promoting survival and growth. L-Gln is a substrate of the glutaminolysis pathway. The first and rate-limiting step of glutaminolysis is the conversion of glutamine to glutamate which is catalyzed by mitochondrial glutaminase (GLS). The glutamate thus formed will be used to fuel the tricarboxylic acid (TCA) cycle. By activating glutaminolysis, cancer cells are able to utilize both aerobic glycolysis (the Warburg effect) and TCA cycle to fulfill their demands for energy and biosynthesis (Pavlova & Thompson, 2016). Imaging glutamine metabolic pathways could provide more insights into tumor biology. PET is a non-invasive and promising imaging modality that has wide application in various disciplines of biology such as oncology (Marcu et al., 2018), neurology (Iaccarino et al., 2017) and cardiology (Li et al., 2014) etc. Many amino acid PET agents containing fluorine-18 (t_½_ = 109.7 min) or carbon-11 (t_½_ = 20.3 min) have been developed for cancer imaging applications. (Sun et al., 2017) In order to study the glutamine metabolic pathways, both the fluorine-18 and carbon-11 versions of glutamine PET agents were developed. (Qu et al., 2012; Qu et al., 2011) In our institution, we have studied the utility of [^18^F](2*S*,4*R*)4-fluoroglutamine and [^11^C]L-glutamine ([^11^C]L-Gln) in various cancers under different interventions (Qu et al., 2012; Zhou et al., 2017). Since [^18^F](2*S*,4*R*)4-fluoroglutamine is shown to be a poor substrate for glutaminase (GLS), we feel that it is important that we study the effect of glutaminase using carbon-11 Gln as the substitute for authentic glutamine molecule. Therefore, obtaining the [^11^C]L-Gln in the enantiomerically enriched form is critical for these studies. The automation of [^18^F](2*S*,4*R*)4-fluoroglutamine has been previously reported (Li et al., 2017). Here we sought to report the synthesis of [^11^C]L-Gln onto a commercially available Synthra HCN Plus synthesis module to improve the convenience, reliability and feasibility of using this radiotracer in preclinical and clinical studies. We along with others have published preliminary results in the abstract previously (Padakanti et al., 2017; Rosenberg et al., 2017; Nasr et al., 2017).

## Methods

### Materials

The standard compounds L-glutamine, D-glutamine, L-glutamic acid, HPLC grade methanol and acetonitrile, 18-crown-6, acetone, trifluoroacetic acid, and concentrated sulfuric acid were purchased from Sigma-Aldrich corporation, USA. Anhydrous DMF, acetonitrile extra dry and cesium bicarbonate, were purchased from Acros Organics, USA. All the gases hydrogen (HY R300), helium (HE R300) and anhydrous ammonia (AM AH200N705) were purchased from Airgas Inc. USA. Deionized Ultra-Filtered Water (DIUF), ethyl ether anhydrous stabilized with BHT were purchased from Fisher scientific, USA. Ethanol 200 proof was purchased from Decon laboratories Inc. PA, USA. All the chemicals were used without any further purification. The seppak cartridges, C18 plus, tC18 vac 3 cc, tC18 plus short and Silica plus were purchased from Waters corporation, USA. The precursor (*S*)-*tert*-butyl-2-((*tert*-butoxycarbonyl)amino)-4-iodobutanoate was purchased from ABX, Advanced biochemical compounds, Germany. Millex GS 0.22 μm filters were from Merck Millipore Ltd. pH strips were from EMD Millipore Corporation, MA, USA. Semi-preparative HPLC column (Macherey-Nagel, VP250/10 Nucleodur C18, HTech, 5 μm was obtained with Synthra HCN Plus synthesis module which was purchased from Synthra GmbH, Hamburg, Germany. QC HPLC column Astec CHIROBIOTIC™ T chiral HPLC column was purchased from Sigma-Aldrich Corporation, USA.

### Instrumentation

[^11^C]CO_2_ was produced on the University of Pennsylvania’s IBA cyclotron by irradiating the gas target of 0.5% O_2_ in N_2_. Approximately 3.6 Ci of [^11^C]CO_2_ was predicted to be produced by a 50 min bombardment of 18 MeV protons using a beam current of 30 μA. The synthesis of [^11^C]L-glutamine including its preparation was performed on a Synthra HCN Plus automated synthesis module. Agilent 1100/1200 series system was used for QC HPLC analysis.

### Preparation

#### Cleaning the synthesis module

**Fig. 1:**
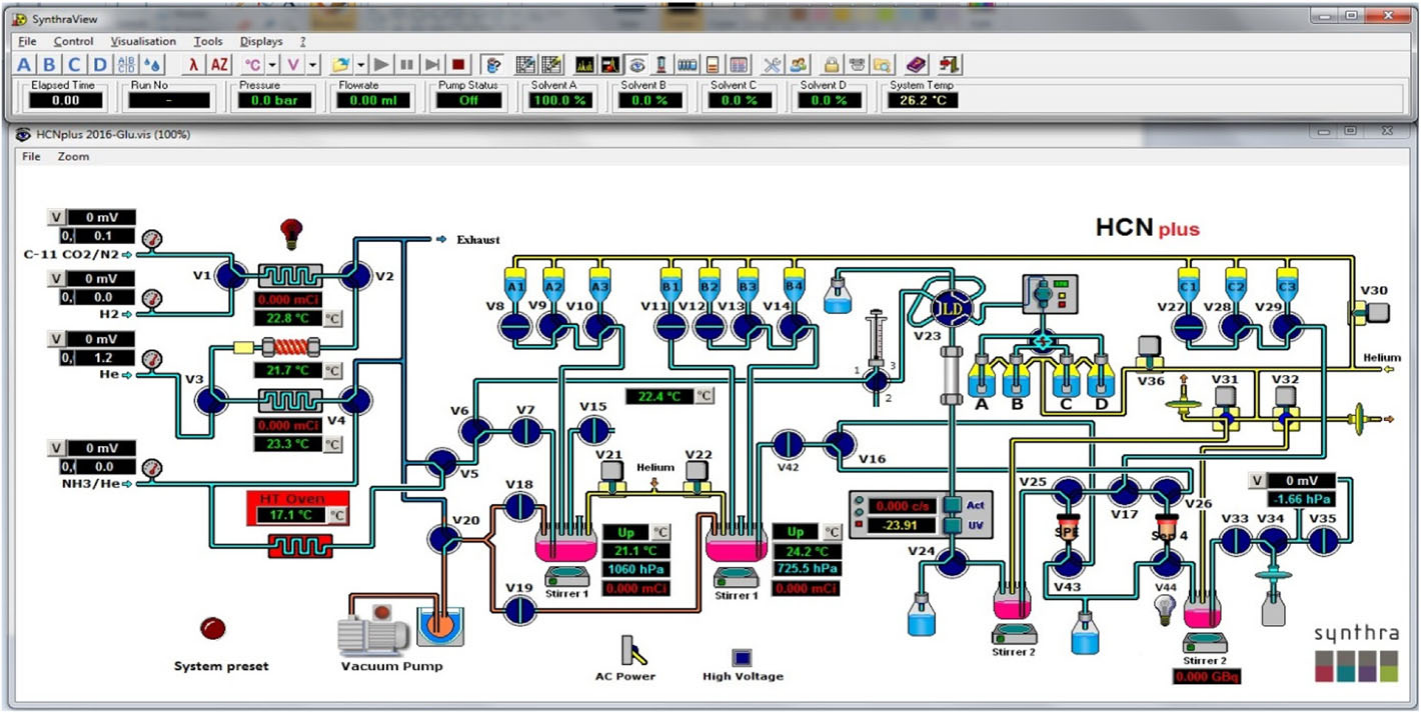
Graphic interface of Synthra HCN Plus automated synthesis module

Cleaning of the synthesis module was performed on Synthra HCN Plus module using the script generated for this purpose. This synthesis module consists of two reaction vessels. Two clean reaction vessels were placed in appropriate positions on the module, the reagent vials A1 through B4 (Fig. 1) were cleaned with acetone, C1 through C3 were cleaned with water followed by ethanol. Acetonitrile and water (DIUF) was used as the HPLC mobile phase. The cleaning program also contains the drying process for all the liquid transfer lines in the system.

#### Azeotropic drying of crown ether and base, and conditioning the system

The system needs to be conditioned prior to the synthesis, on the day of production. While the system is being conditioned, the mixture of 18-crown-6 (18-C-6) and cesium bicarbonate were azeotropically dried. It is critical that the reaction conditions are anhydrous and not too basic to obtain the final product [^11^C]L-glutamine in high enantiomeric purity. Therefore, conditioning of the synthesis module process was combined with the azeotropic drying of 18-C-6 and cesium bicarbonate using acetonitrile. Vials V1, V2 and V3 were filled with 1.0 mL, 0.5 mL and 0.5 mL of acetonitrile respectively, and reaction vessel 1 was filled with 18-C-6 (8 mg in 900 uL of acetonitrile from the stock solution) and cesium bicarbonate (3 mg in 150 uL of DIUF from the stock solution). Clean and dry reaction vessel 2 was also placed in appropriate position on the module. The conditioning of the system includes flushing the [^11^C]CO_2_ trap loop with hydrogen while heating the [^11^C]CO_2_ trap temperature to 150 °C and stopping the heating. The hydrogen flush was continued and diverted to a nickel column heated to 420 °C, and a methane trap (Carboxen®) heated to 200 °C to exhaust. While this part of the module is flushing with hydrogen gas, platinum column was flushed with anhydrous ammonia. Once this process is complete, helium is flushed through platinum column heated to 990 °C. The mixture of crown ether and cesium bicarbonate solution was dried azeotropically with acetonitrile under vacuum and helium flow. The helium flow through the platinum column was diverted to the reaction vessel 1 to thoroughly dry the mixture until the end of conditioning step, approximately 150 min.

#### Automated radiochemical synthesis of [^11^C]L-glutamine

**Scheme 1:**
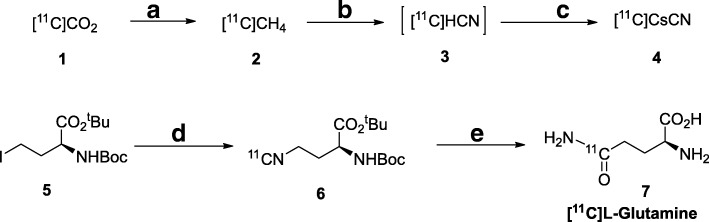
Radiochemical Synthesis of [^11^C]L-glutamine 7

The synthesis was performed as shown in Scheme 1. [^11^C]CO_2_ generated from the cyclotron was delivered to the synthesis module where the [^11^C]CO_2_ was trapped cryogenically by cooling the trap to approximately − 180 °C using liquid nitrogen. The [^11^C]CO_2_ was then converted to [^11^C]CH_4_ by passing it using hydrogen flow over the nickel column heated to 420 °C. [^11^C]CH_4_ was trapped on a Carboxen® column cooled to − 120 °C. Using the controlled helium flow (10 mL/min) the [^11^C]CH_4_ trapped on methane trap was passed over the platinum column heated to 990 °C while mixing with anhydrous ammonia (flow: 10 mL/min) into the reaction vessel 1. In this process, the [^11^C]CH_4_ was converted to [^11^C] cyanide, which was trapped in the reaction vessel containing azeotropically dried 18-C-6 and cesium bicarbonate in 0.25 mL anhydrous DMF. The excess ammonia was sparged off by bubbling the mixture with helium (20 mL/min) for additional 1 min. The precursor **5** (2–4 mg) in 0.25 mL of anhydrous DMF was added via reagent vial A2 into the reaction vessel 1. The mixture was heated at 90 °C for 8 min and quenched by adding 1.0 mL of with HPLC purification mobile phase via reagent vial A1. The reaction mixture was loaded onto HPLC, and the intermediate 6 was collected at around 7 min into the flask containing 35 mL of DIUF water. The intermediate was trapped on a C18 sep-pak plus cartridge, and washed with additional DIUF water via reagent vial C1. The intermediate 6 was eluted into the reaction vial 2 using acetonitrile from vial C3, and dried under vacuum in combination with helium flow. This drying step was manually controlled to ensure the drying process is complete, as visualized in the camera equipped in the hot cell. Concentrated sulfuric acid (from vial B4) and trifluoroacetic acid (from vial B1) were then added to the reaction vessel 2 containing dried intermediate 6. The reaction mixture was heated at 80 °C for 5 min. The mixture was cooled to 40 °C and diluted with 2.5 mL of ethyl ether (from vial B2) and transferred onto silica plus sep-pak, and the sep-pak was washed with additional 10 mL ethyl ether (from vial B3). The sep-pak was dried by passing helium through. The final product trapped on the silica plus sep-pak was collected using 4 mL (from vial C2) of sterile water for injection (SWI) into the product collection flask containing 0.6 mL of phosphate buffer and 4.0 mL of SWI. The mixture was sparged with helium for additional 5 min to sparge off excess ether. The contents in the product collection flask were transferred into final product vial via sterilization filter to obtain the final product [^11^C]L-glutamine. Due to the low UV absorbance of glutamine, we had used an indirect method for determining the molar activity of [^11^C]L-glutamine by converting the glutamine to its Fmoc derivative, an adaption of the method published by Qu et al. (Gleede et al., 2015)

#### Quality control

**Fig. 2:**
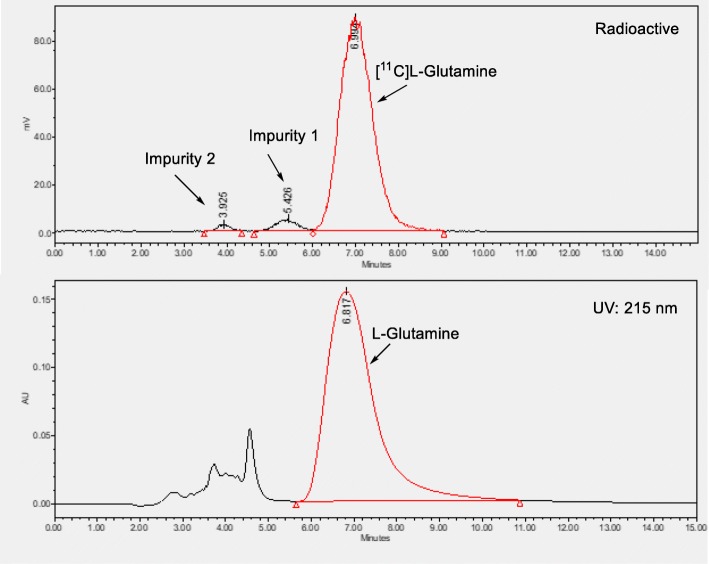
HPLC chromatograms of C-11 glutamin

The HPLC analysis of the final product was performed on an Agilent HPLC unit and the data was collected and analyzed using Waters Empower software. Astec CHIROBIOTIC™ T HPLC column 25 cm × 4.6 mm, 5 μm was used for analytical purpose. Final product was sterile filtered using 0.22 μm GS filter. The pH of the final product was approximately 6.0. The final product was obtained with the radiochemical purity of > 90% with less than 5% of [^11^C]D-glutamine (Fig. 2). The other radiochemical impurity was predicted to be the unhydrolyzed, deprotected intermediate and/or [^11^C] glutamic acid. The molar activity was greater than 7000 mCi/μmol at the EOB.

## Results and discussion

**Fig. 3:**
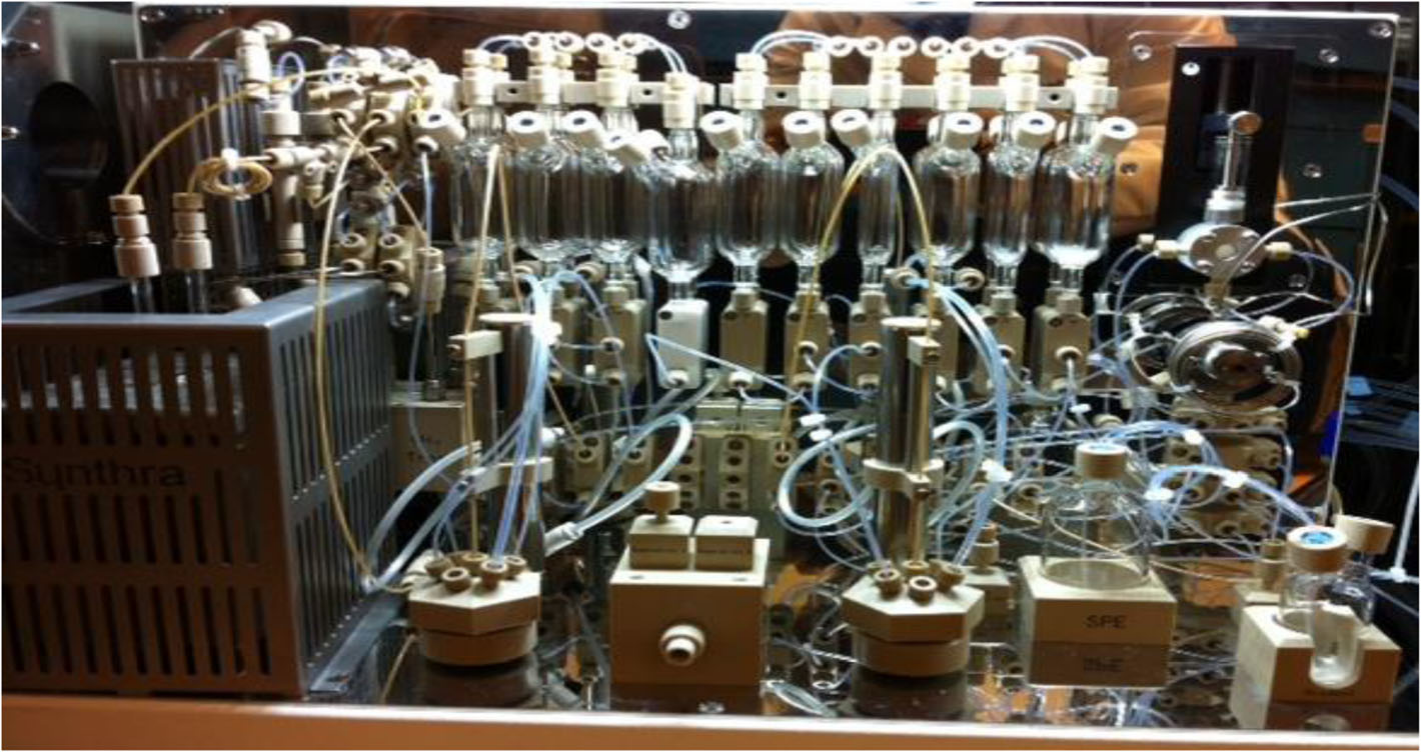
Synthra HCN Plus synthesis module

The synthesis script was initially developed based on the radiolabeling conditions from our manual runs. It was later modified following the literature protocol (Gleede et al., 2015). Complete synthesis including azeotropic drying of the crown ether and cesium bicarbonate was accomplished on a single synthesis module (Fig. 3). Several test runs were performed to optimize the automation process.

Radiosynthesis of [^11^C]L-glutamine needs to be obtained in an enatiopure form. Therefore the synthesis began with the commercially available enantiopure precursor, (*S*)-*tert*-butyl-2-((*tert*-butoxycarbonyl)amino)-4-iodobutanoate **5**. Synthesis was performed on Synthra HCN Plus automated synthesis module (Fig. 3), and the process was remotely observed on a laptop using Graphic User Interface (Fig. 1). The critical parts of the synthesis are anhydrous conditions and optimal ammonia in the reaction.

In Part I of the synthesis, during the conversion of [^11^C]CH_4_ to [^11^C] CsCN, mixture of helium and ammonia gases was used. The amount of ammonia used is very critical in obtaining the enantioenrichment in the final product. Racemized product was observed if excess ammonia existed at the presence of moisture in the first [^11^C] nitrile insertion step, and low yield of [^11^C] CsCN was observed if less ammonia was used. Therefore, the reaction mixture was bubbled with helium to remove excess ammonia. Azeotropic drying of the crown ether and base was performed to remove moisture prior to the reaction. The use of unconventional cesium bicarbonate was due to the reported method suggesting [^11^C] CsCN gave the best result (Gleede et al., 2015). The precursor 5 in DMF was added to reaction vessel 1 and heated. After the [^11^C] nitrile insertion step, the resultant [^11^C] nitrile intermediate 6 was purified on a semi-preparative HPLC to remove unreacted [^11^C] CsCN and other potential impurities. The intermediate 6 needs to be dried before it is treated with mixture of acids in the second step. Considering the size and geometry of the reaction vessel equipped with the synthesis module, it was observed that using less than 1.5 mL of acetonitrile to elute the intermediate into the second reaction vessel is optimal. Therefore, attempts were made to evaluate the trapping of the intermediate 6 on various solid-phase extraction cartridges with different material and sizes of packing material, such as tC18 vac 3 cc, tC18 light, tC18 plus short, and C18 plus sep-pak. Of the sep-paks used, only the C18 sep-pak plus was able to trap the intermediate 6 efficiently. In order to efficiently elute the intermediate **6** from C18 plus sep-pak, it was observed that at least 1.4 mL of acetonitrile was needed. If excess acetonitrile was used, part of the solution was transferred into the drying lines. If less acetonitrile was used, the radioactivity recovery was low.

Azeotropic drying of the intermediate **6** at this stage was also critical. The drying of the intermediate takes approximately 10–15 min. It was observed that incomplete drying will lead to some hydrolysis to give [^11^C] glutamic acid as an impurity. After drying, the second step (deprotection/hydrolysis) was performed in the presence of mixture of strong acids, concentrated sulfuric acid and trifluoroacetic acid at 80 °C for 5 min. The final product after the reaction was diluted with diethyl ether and passed through silica plus sep-pak. The silica plus sep-pak was washed with additional 10 mL of diethyl ether. The resultant [^11^C]L-glutamine trapped on silica sep-pak was collected by eluting with 4 mL of SWI into a buffer containing phosphoric acid to adjust the pH of the final solution between 4.5 and 7 (mostly ~ 6). This pH adjusted product was sparged with helium to remove diethyl ether and sterile filtered using the GS filter into the final product vial. If the concentrated sulfuric acid and trifluoroacetic acid bottles were opened many times (5–10 times), we observed formation of impurities. Over all, the possible radiochemical impurities that can be predicted from the synthesis of [^11^C]L-glutamine are [^11^C]D-glutamine, [^11^C]L-glutamic acid, [^11^C]**6** and deprotected nitrile analog of **6** (Impurity 1, Fig. 2) of [^11^C]**6**. The retention time of all these predicted compounds were below 10 min on Astec CHIROBIOTIC™ T QC HPLC column using 80:20 methanol:water as the mobile phase at a flow rate of 1.0 mL/min. The approximate retention times (Rt) of D-glutamine, impurity 1 (Fig. 2, based on prediction), intermediate 6 and L-glutamic acid are 9, 5, 3.8 and 3.5 min, respectively.

We had investigated the possible side reactions for the formation of these impurities. Based on the impurity observed, we attempted to predict the cause. [^11^C] D-glutamine (Rt 9 min) was observed if the reaction condition was too basic in combination with moisture, in the [^11^C] nitrile insertion step. This happened when the drying of the crown ether and cesium bicarbonate was not thorough in combination with excess ammonia. The impurity 1 (Rt 5 min) was observed if the second step reaction (acidic conditions) was not complete either due to splashing of the intermediate 6 to the top of the vial or other reasons. The impurity (6) (Rt 3.8 min) was observed if there was any trace of 6 left in the lines when transferring from C18 sep-pak plus to reaction vessel 2. L-glutamic acid (Rt 3.5 min) was observed if the azeotropic drying of the intermediate 6 was not completed resulting in opportunity of further hydrolysis. If the desired product was observed as a minor radioactivity peak, it is an indication that the sulfuric acid and trifluoroacetic acid needs to be replaced.

Another issue that was faced was incompatibility of manifolds on the synthesis module with the strong acids. During the initial translation into automation, the acids were premixed and loaded into the reagent vial. When the premixed acid was used, the peek manifold used for the transfer of acids were degraded and clogged. Attempts were then made to add the acids separately into the reaction vial, but even this did not solve the issue. In order to avoid this issue, custom made teflon manifolds for the reagent vials for both sulfuric acid and trifluoroacetic acid were used. Although this solved the issue of the clog in the manifold, the transfer line from reaction vessel 2 to valve 42 (transfer connection to silica sep-pak) started to degrade at the reaction vessel end. Therefore, we replaced the peek tubing with tefzel tubing, which is replaced after every run to avoid any corrosion. Tefzel and FEP tubing materials were also found to be compatible based on visual inspection.

After all the above optimization process, we were able to successfully and reliably (*n* = 4 consecutive syntheses) synthesize [^11^C]L-glutamine. The synthesis time from the end of [^11^C] cyanide trapping into the reaction vessel was approximately 60 min. Starting with approximately 1.0 Ci of [^11^C] cyanide, 47–77 mCi of final [^11^C]L-glutamine was obtained after sterile filtration. The decay corrected yield from the [^11^C] cyanide trapping into the reaction vessel was 43–52% (n = 4).

## Conclusion

In conclusion, successful automation of the synthesis of [^11^C]L-glutamine on a Synthra HCN Plus synthesis module was accomplished. Synthesis was reliable and reproducible (n = 4 consecutive syntheses). The final product was obtained in radiochemical purity was > 90%. This process can be readily adapted for future clinical research use.

## Abbreviations

CH_4_: Methane
CO_2_: Carbon dioxide
CsCN: Cesium cyanide
DIUF: Deionized Ultr-Filtered
DMF: N,N-Dimethylformamide
Gln: Glutamine
HPLC: High Performance Liquid Chromatography
PET: Positron Emission Tomography
SWI: Sterile Water for Injection
